# Effects of High CO_2_ on Growth and Metabolism of Arabidopsis Seedlings During Growth with a Constantly Limited Supply of Nitrogen

**DOI:** 10.1093/pcp/pct186

**Published:** 2014-01-18

**Authors:** Nobuyuki Takatani, Takuro Ito, Takatoshi Kiba, Marie Mori, Tetsuro Miyamoto, Shin-ichi Maeda, Tatsuo Omata

**Affiliations:** ^1^Graduate School of Bioagricultural Sciences, Nagoya University, Nagoya, 464-8601 Japan; ^2^Institute for Advanced Biosciences, Keio University, 246-2 Mizukami, Kakuganji, Tsuruoka, Yamagata, 997-0052 Japan; ^3^Systems Biology Program, Graduate School of Media and Governance, Keio University, Fujisawa, 252-8520 Japan; ^4^PRESTO, Japan Science and Technology Agency, Kawaguchi, 332-0012 Japan; ^5^RIKEN Center for Sustainable Resource Science, Yokohama, 230-0045 Japan

**Keywords:** Arabidopsis, High-CO_2_ response, Metabolome, Nitrogen limitation

## Abstract

Elevated CO_2_ has been reported to stimulate plant growth under nitrogen-sufficient conditions, but the effects of CO_2_ on growth in a constantly nitrogen-limited state, which is relevant to most natural habitats of plants, remain unclear. Here, we maintained Arabidopsis seedlings under such conditions by growing a mutant with reduced nitrate uptake activity on a medium containing nitrate as the sole nitrogen source. Under nitrogen-sufficient conditions (i.e. in the presence of ammonium), growth of shoots and roots of both the wild type (WT) and the mutant was increased approximately 2-fold by elevated CO_2_. Growth stimulation of shoots and roots by elevated CO_2_ was observed in the WT growing with nitrate as the sole nitrogen source, but in the mutant grown with nitrate, the high-CO_2_ conditions stimulated only the growth of roots. In the mutant, elevated CO_2_ caused well-known symptoms of nitrogen-starved plants, including decreased shoot/root ratio, reduced nitrate content and accumulation of anthocyanin, but also had an increased Chl content in the shoot, which was contradictory to the known effect of nitrogen depletion. A high-CO_2_-responsive change specific to the mutant was not observed in the levels of the major metabolites, although CO_2_ responses were observed in the WT and the mutant. These results indicated that elevated CO_2_ causes nitrogen limitation in the seedlings grown with a constantly limited supply of nitrogen, but the Chl content and the root biomass of the plant increase to enhance the activities of both photosynthesis and nitrogen uptake, while maintaining normal metabolism and response to high CO_2_.

## Introduction

Atmospheric CO_2_ concentration has risen from about 280 p.p.m. in pre-industrial times to 400 p.p.m., and is predicted to reach 530–970 p.p.m. by the end of the 21st century (IPCC 2007). Elevated CO_2_ leads to increased rates of carboxylation of ribulose-1,5-bisphosphate relative to the rates of the oxygenation reaction in the C_3_ plant, resulting in a higher net rate of photosynthesis and stimulated plant growth (Stitt 1991, Drake et al. 1997). However, long-term exposure to elevated CO_2_ leads to reduction in Rubisco content and the maximum rate of the carboxylation reaction, a phenomenon known as the down-regulation of photosynthesis, which is thought to represent an acclimation process to high-CO_2_ conditions (Stitt and Krapp 1999, Long et al. 2004). The response to elevated CO_2_ is affected by the nitrogen supply (Cheng et al. 1998, Stitt and Krapp 1999). The down-regulation of photosynthesis has been reported to be more pronounced in nitrogen-limited plants than in well-fertilized plants (Stitt and Krapp 1999, Sun et al. 2002, Sanz-Sáez et al. 2010). Moreover, transfer of Arabidopsis plants to a high-CO_2_ environment was shown to induce the responses typical of nitrogen-starved plants (Li et al. 2008). These observations raise a question about the physiological state of the plants growing in a high-CO_2_ environment with a constantly limited supply of nitrogen, because the latter is relevant to most natural habitats of plants. It is, however, not simple to maintain a constantly nitrogen-limited state in an experimental condition, since the plants will soon deplete the nitrogen from the media containing small amounts of nitrogen and will be starved of nitrogen, and it is not easy to control the nitrogen supply through irrigation (Tschoep et al. 2009). Chemostat culture is commonly used to grow the cells of microorganisms with a constantly limited supply of a nutrient, but it is not practical to apply this technique to plants. As an alternative approach to the nutrient-limited conditions, a mutant defective in active transport of nitrate was used in the cyanobacterium *Synechococcus elongatus* PCC 7942 (Aichi et al. 2004). In media containing high concentrations of nitrate, the mutant grows slowly, depending on the passive entrance of nitrate into the cell, and shows a phenotype specific to the constantly nitrogen-limited cells. In this study, we attempted to adapt this approach to *Arabidopsis thaliana*.

In *A. thaliana*, five nitrate transporters, NRT1.1 (CHL1), NRT1.2, NRT2.1, NRT2.2 and NRT2.4, function in the root (Tsay et al. 1993, Wang et al. 1998, Huang et al. 1999, Liu et al. 1999, Cerezo et al. 2001, Filleur et al. 2001, Orsel et al. 2004, Li et al. 2007, Kiba et al. 2012). NRT1.1 has been shown to display dual-affinity transport activity depending on phosphorylation of the Thr101 residue (Liu and Tsay 2003). Although the *NRT1.1*-deficient mutants were shown to have a reduced nitrate uptake capacity in the low and/or high concentration range (Tsay et al. 1993, Touraine and Glass 1997, Liu and Tsay 2003, Muños et al. 2004), they are not useful for this study because of a function of NRT1.1 as a nitrate sensor (Ho et al. 2009). NRT1.2 is a low-affinity carrier (Huang et al. 1999), which was recently reported to mediate cellular ABA uptake (Kanno et al. 2012). NRT2.1, NRT2.2 and NRT2.4 are high-affinity nitrate carriers (Wang et al. 1998, Cerezo et al. 2001, Filleur et al. 2001, Orsel et al. 2004, Li et al. 2007, Kiba et al. 2012), whose absence in triple knockout (TKO) mutants of *NRT2.1*, *NRT2.2* and *NRT2.4* results in an approximately 75% decrease in growth in the presence of 0.5 mM nitrate with no obvious side effects on plant morphology and development (Kiba et al. 2012). We therefore chose an *NRT2* TKO mutant as the plant material. As the first step to understanding the plant responses to a high-CO_2_ and low-nitrogen environment, we established in this study a high-CO_2_-induced, constantly nitrogen-limited growth state in *A. thaliana* seedlings. The unique phenotype of the seedlings is discussed in comparison with that observed in nitrogen-starved plants.

## Results

### Differential effects of elevated CO_2_ on growth of WT and TKO_2-1/2/4 mutant seedlings on nitrate-containing medium

**Fig. 1:**
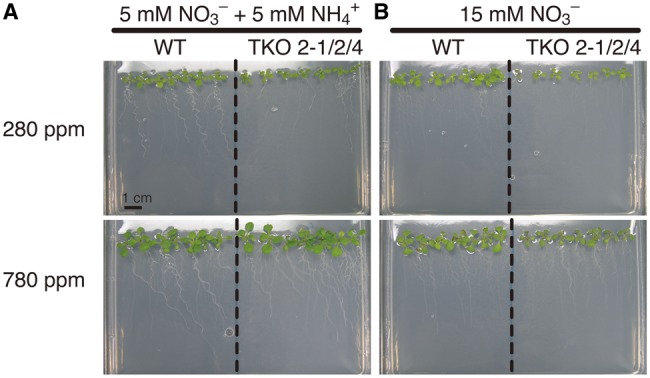
Effects of elevated CO_2_ level on growth of the seedling of the WT and the *NRT2* TKO mutant. Plants were grown on solid medium containing 5 mM 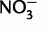 and 5 mM 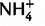 (N5A5 medium) or 15 mM 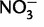 (N15 medium) under low CO_2_ (280 p.p.m.) or elevated CO_2_ (780 p.p.m.) for 10 d.

**Fig. 2:**
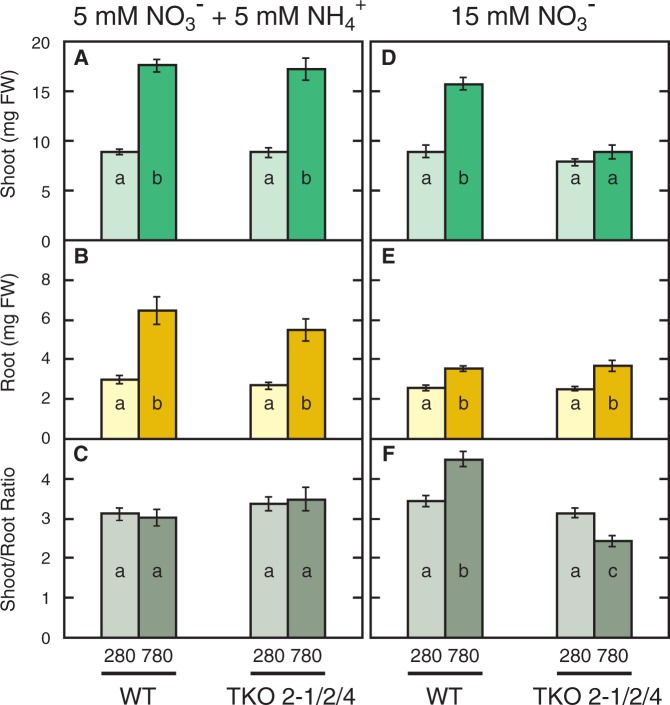
Effects of elevated CO_2_ on shoot (A, D) and root (B, E) fresh weight and the shoot/root ratio (C, F) of the WT and the TKO_2-1/2/4 mutant. Seedlings were grown on the N5A5 medium (A, B, C) or the N15 medium (D, E, F) under low CO_2_ (280 p.p.m.) or elevated CO_2_ (780 p.p.m.) for 10 d. Data shown are the means ± SE from three experimental replicates (*n* = 15, five plants per one experimental replicate). Different letters denote significant differences from Scheffe’s multiple comparison test (*P* < 0.05) conducted for each nitrogen condition.

To assess the effects of elevated CO_2_ on the plants under different levels of nitrogen supply, we used two Arabidopsis strains (the WT and a TKO mutant of *NRT2.1*, *NRT2.2* and *NRT2.4*) and two nitrogen conditions (5 mM 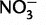 plus 5 mM 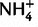, and 15 mM 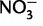). The TKO mutant used was the ‘*nrt2.1-2 nrt2.4-1*’ strain generated previously by Kiba et al. (2012) (hereafter referred to as TKO_2-1/2/4). Given that Arabidopsis roots have a much higher capacity for ammonium uptake than for nitrate uptake (Gazzarrini et al. 1999), it was assumed that both the WT and the TKO_2-1/2/4 mutant can assimilate sufficient N on the medium containing 5 mM each of 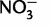 and 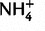 (hereafter referred to as the N5A5 medium), whereas growth of the TKO_2-1/2/4 mutant but not of the WT could be limited on the medium containing 15 mM 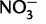 as the sole source of nitrogen (hereafter referred to as the N15 medium) due to the reduced nitrate transport activity of the mutant. Seedlings were grown under low CO_2_ (280 p.p.m.) and high CO_2_ (780 p.p.m.) to compare their growth. On N5A5 medium, growth of the WT and TKO_2-1/2/4 mutant and the effects of elevated CO_2_ thereon were essentially the same, showing stimulation of growth of shoots and roots under the high-CO_2_ conditions (Fig. 1A). Seedling growth was also stimulated under the high-CO_2_ condition on the N15 medium in the WT (Fig. 1B). The TKO_2-1/2/4 mutant, in contrast, showed no apparent response to elevated CO_2_ on the N15 medium (Fig. 1B). The fresh weight of shoots and roots was measured to determine the effects of CO_2_ quantitatively (Fig. 2). Elevated CO_2_ increased the growth of shoots and roots 2.0-fold in the WT and TKO_2-1/2/4 mutant on the medium containing 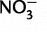 and 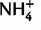 (Fig. 2A, B). As a result, elevated CO_2_ did not affect the shoot/root ratio in either the WT or TKO_2-1/2/4 mutant (Fig. 2C). In the medium containing 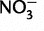 as the sole nitrogen source, elevated CO_2_ increased the fresh weight of the shoots and roots of the WT 1.8 - and 1.4-fold, respectively (Fig. 2D, E). In contrast, the TKO_2-1/2/4 mutant showed no significant increase in the shoot fresh weight in response to elevated CO_2_, while the root fresh weight was increased by 1.4-fold (Fig. 2D, E). Elevated CO_2_ thus significantly decreased the shoot/root ratio in the TKO_2-1/2/4 mutant, while significantly increasing it in the WT (Fig. 2F).

### Nitrate content in the WT and the TKO_2-1/2/4 mutant

**Fig. 3:**
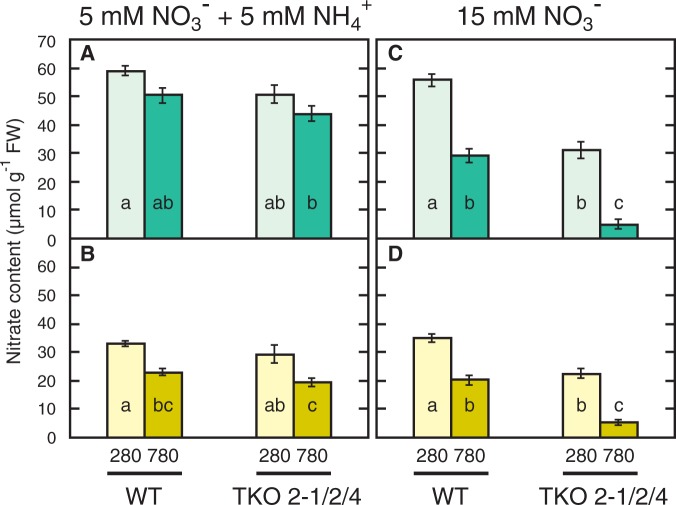
Effects of elevated CO_2_ on nitrate content in shoots (A, C) and roots (B, D) of the WT and the *NRT2* TKO mutant. Seedlings were grown on the N5A5 medium (A, B) or the N15 medium (C, D) under low CO_2_ (280 p.p.m.) or elevated CO_2_ (780 p.p.m.) for 10 d. Means ± SE of the data from three experimental replicates are shown (*n* = 15, five plants per one experimental replicate). Different letters denote significant differences from a Scheffe’s multiple comparison test (*P* < 0.05) conducted for each nitrogen condition.

When grown on the medium containing both 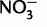 and 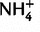, the WT and the TKO_2-1/2/4 mutant growing under the low-CO_2_ conditions accumulated large amounts (50–60 µmol g^−1^ FW and 30–35 µmol g^−1^ FW in shoots and roots, respectively) of nitrate (Fig. 3A, B). Elevated CO_2_ slightly decreased the nitrate content, i.e. by 15% in the shoot and by 35% in the root, in both the WT and TKO_2-1/2/4 mutant. The accumulation of nitrate in the TKO_2-1/2/4 mutant is presumably due to the activity of AtNRT1.1 and AtNRT1.2. Even though the TKO_2-1/2/4 mutant has much lower NRT activity as compared with the WT (Kiba et al. 2012), the present results indicate that it is high enough to maintain the nitrate pool under the conditions where ammonium is used as the primary source of nitrogen. On the N15 medium, the WT grown under the low-CO_2_ conditions accumulated nitrate to the same level as observed on the N5A5 medium. Elevated CO_2_ decreased the nitrate content by 50% in the shoot and by 40% in the root. The decrease in nitrate content is ascribed to consumption of larger amounts of nitrate because of the stimulation of growth by elevated CO_2_. Under the high-CO_2_ conditions, the nitrate levels in shoots and roots corresponded to approximately 30 and 20 mM, respectively, indicating that the seedlings still maintained a large pool of inorganic nitrogen. Nitrate contents of the shoot and root of the TKO_2-1/2/4 mutant grown on the N15 medium under the low-CO_2_ conditions were much lower than the corresponding WT levels (55% and 65% in the shoot and the root, respectively) and were similar to the wild-type levels under 780 p.p.m. CO_2_. The nitrate contents in the TKO_2-1/2/4 mutant were decreased to very low levels when grown under a high-CO_2_ condition. These results showed that the limitation of shoot growth in the TKO_2-1/2/4 mutant under the high-CO_2_ conditions on the N15 medium was caused by the limited supply of nitrate.

### Anthocyanin content in the WT and the TKO_2-1/2/4 mutant

**Fig. 4:**
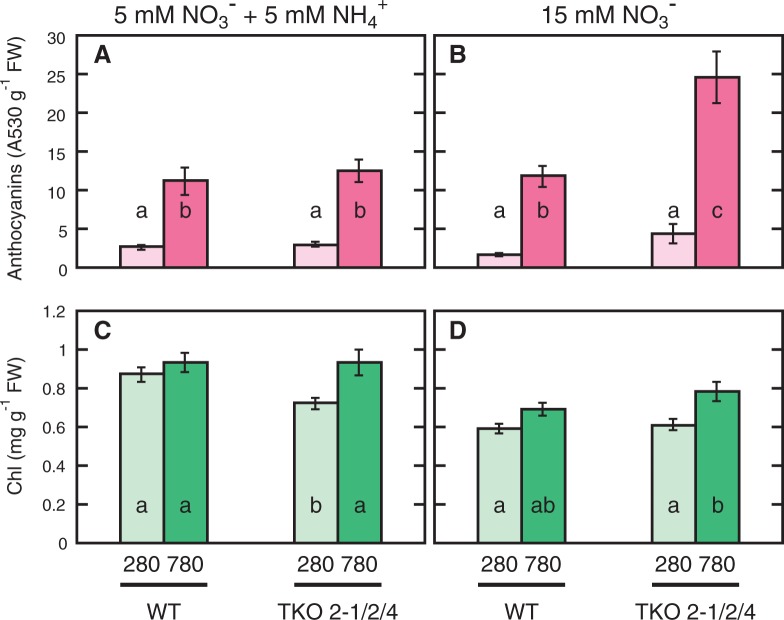
Effects of elevated CO_2_ on the anthocyanin (A, B) and Chl (C, D) contents in the WT and the *NRT2* TKO mutant. Seedlings were grown on the N5A5 medium (A, C) or the N15 medium (B, D) under low CO_2_ (280 p.p.m.) or elevated CO_2_ (780 p.p.m.) for 10 d. Means ± SE of the data from 6–9 experimental replicates are shown (*n* = 30–45, five plants per one experimental replicate). Different letters denote significant differences from a Scheffe’s multiple comparison test (*P* < 0.05) conducted for each nitrogen condition.

Anthocyanins are accumulated in the leaves in response to the higher carbon/nitrogen balance (Martin et al. 2002). Elevated CO_2_ increased the anthocyanin content in the shoot of both strains regardless of the nitrogen conditions (Fig. 4A, B), indicating that the plants grown under 780 p.p.m. CO_2_ did respond to increased carbon availability. In particular, the TKO_2-1/2/4 mutant growing under an elevated CO_2_ condition with nitrate as the sole nitrogen source accumulated large amounts of anthocyanins. This supported the notion above that the TKO_2-1/2/4 mutant growing under a high-CO_2_ condition is experiencing the stress of nitrogen limitation.

### Chl content in the shoots of the WT and the TKO_2-1/2/4 mutant

Chl content in the shoot was higher in the seedlings of the WT grown on the N5A5 medium than on the N15 medium, and elevated CO_2_ only slightly increased the Chl content under both nitrogen conditions (Fig. 4C, D). In contrast, elevated CO_2_ increased the Chl content in the *NRT2* TKO mutant by 25% on the N5A5 medium and 28% on the N15 medium. The high-CO_2_-induced increase of Chl content in the TKO_2-1/2/4 mutant on the N15 medium was unexpected, because Chl content is known to be reduced under nitrogen-limited conditions (Peng et al. 2007). Thus, the response of the mutant to the constantly nitrogen-limited condition is distinct from the plant’s response to conventional nitrogen stress.

### Relative content of carbon and nitrogen in the shoot of the WT and the TKO_2-1/2/4 mutant

**Fig. 5:**
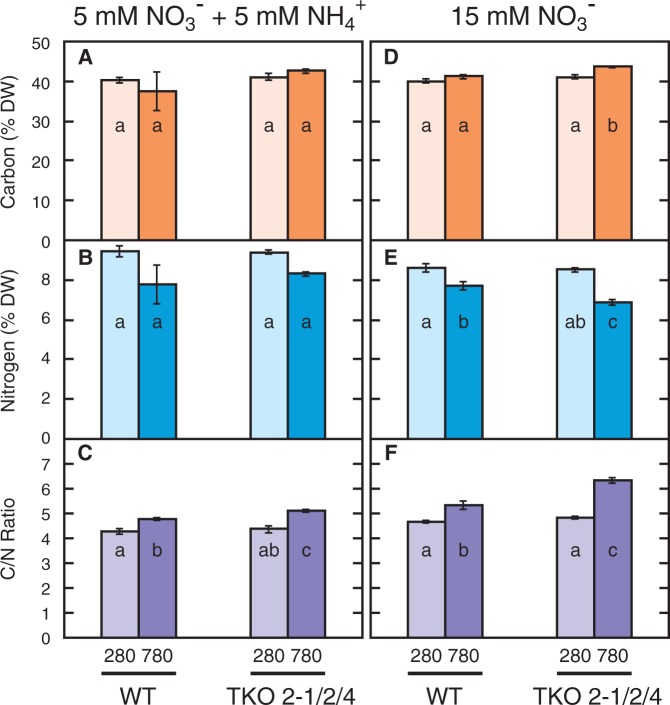
Effects of elevated CO_2_ on the carbon (A, D) and nitrogen (B, E) contents and the C/N ratio (C, F) in the WT and the TKO_2-1/2/4 mutant. Plants were grown on the N5A5 medium (A–C) or the N15 medium (D–F) under low CO_2_ (280 p.p.m.) or elevated CO_2_ (780 p.p.m.) for 10 d. Data shown are the means ± SE from three experimental replicates (*n* = 3). Different letters denote significant differences from a Scheffe’s multiple comparison test (*P* < 0.05) conducted for each nitrogen condition.

Elevated CO_2_ did not affect the carbon content per dry weight of the shoot of either the WT or TKO_2-1/2/4 mutant under either nitrogen condition (Fig. 5A, D), but decreased the nitrogen content of the shoot in both strains under both nitrogen conditions (Fig. 5B, E). The decline in the nitrogen content resulted in a significant increase in the C/N ratio under elevated CO_2_ (Fig. 5C, F), particularly in the TKO_2-1/2/4 mutant growing on the N15 medium.

### Effects of nitrogen deficiency on growth and Chl and anthocyanin contents of the WT and the TKO_2-1/2/4 mutant

**Fig. 6:**
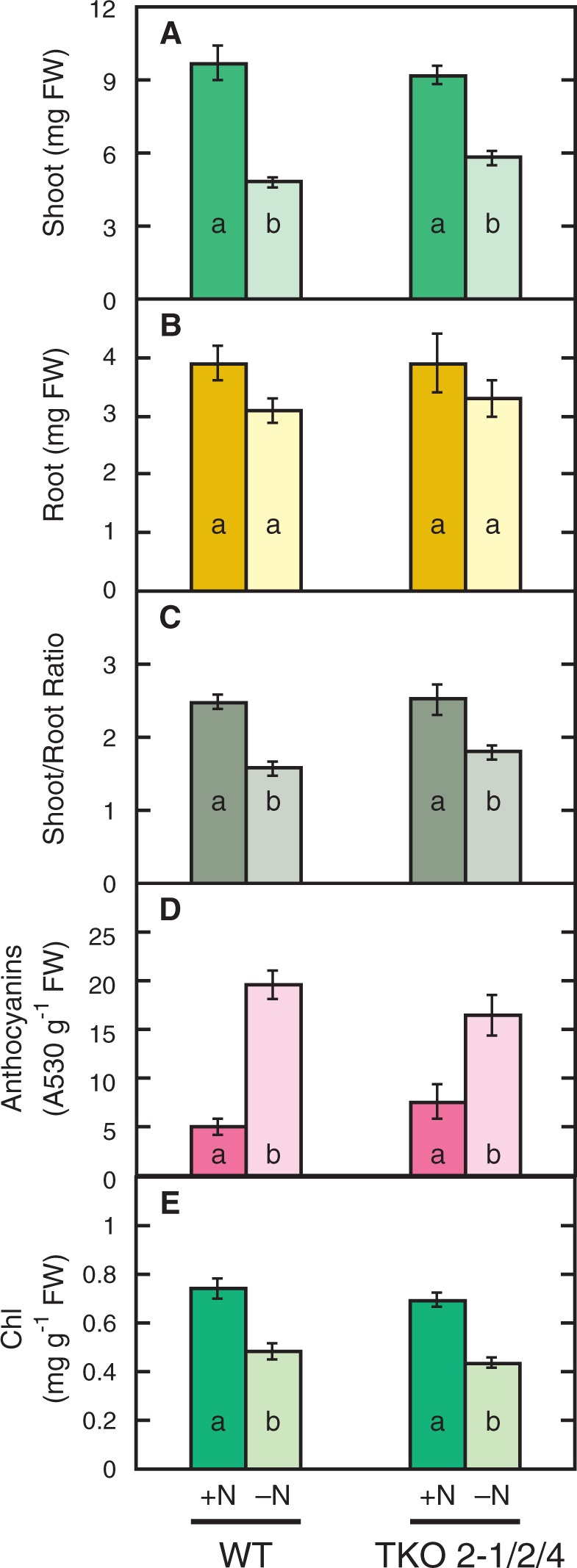
Effects of nitrogen deficiency on shoot (A) and root (B) fresh weight, the shoot/root ratio (C), and anthocyanin (D) and Chl (E) contents of the WT and the *NRT2* TKO mutant. Seven-day-old plants grown on the N5A5 medium were transferred to fresh N5A5 medium (+N) or to a medium containing no nitrogen sources (–N) and grown for 3 d under ambient CO_2_. Means ± SE of the data from three experimental replicates (*n* = 12, four plants per one experimental replicate) are shown in A, B, C and E, and those from five plants are shown for D. Different letters denote significant differences from Scheffe’s multiple comparison test (*P* < 0.05).

**Fig. 7:**
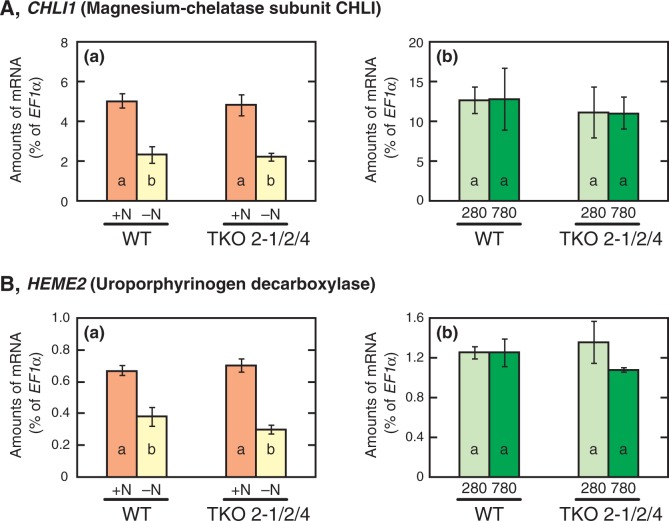
Changes of the transcript levels of *CHLI1* (A) and *HEME2* (B) in the WT and the *NRT2* TKO mutant in response to nitrogen deficiency (a) and the high-CO_2_ treatment on the N15 medium (b). (a) Seven-day-old plants grown on the N5A5 medium were transferred to fresh N5A5 medium (+N) or to a medium containing no nitrogen sources (–N) and grown for 3 d under ambient CO_2_. (b) Plants were grown on the N15 medium under low CO_2_ (280 p.p.m.) or elevated CO_2_ (780 p.p.m.) for 10 d. Data shown are the means ± SE from three experimental replicates (*n* = 3). Different letters denote significant differences from a Scheffe’s multiple comparison test (*P* < 0.05) conducted for each set of data.

The low nitrate content, high anthocyanin content and the high C/N ratio in the TKO_2-1/2/4 seedlings grown under the high-CO_2_ conditions with 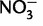 as the nitrogen source were considered to reflect the nitrogen-limited state of the plant. The higher content of Chl under these conditions, however, was unusual as a response to a limited nitrogen supply. When the 7-day-old WT and mutant plants growing on the N5A5 medium (nitrogen-sufficient, +N conditions) were transferred to the medium without a nitrogen source and incubated for 3 d (nitrogen-deficient, –N conditions) under ambient CO_2_ conditions, shoot growth was decreased by 40% as compared with the plants grown for 10 d under the +N conditions, whereas root growth was only slightly decreased (Fig. 6A, B). As a result, the shoot/root ratio of both strains was significantly decreased under the –N conditions compared with the +N conditions (Fig. 6C). The decrease in shoot/root ratio was distinct from that observed in the response of the *NRT2* TKO mutant to high CO_2_ on 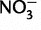-containing medium, because the latter was caused by the increase in root fresh weight (Fig. 2). The anthocyanin content was markedly increased under the –N conditions compared with the +N conditions in both strains (Fig. 6D). The Chl content of both strains was significantly decreased under the –N conditions (Fig. 6E). In accordance with the previous report that expression of the genes involved in Chl biosynthesis is decreased under nitrogen-deficient conditions (Peng et al. 2007), levels of expression of *CHLI1*, encoding a magnesium chelatase subunit CHLI, and *HEME2*, encoding a uroporphyrinogen decarboxylase, were decreased by 50–60% by nitrogen deficiency in both the WT and the TKO_2-1/2/4 mutant (Fig. 7A, B, panels a). In contrast, neither the mutant nor the WT showed changes in the expression levels of these genes in response to high CO_2_ on the 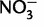-containing medium (Fig. 7A, B, panels b). These results confirmed that the high-CO_2_-induced, constantly nitrogen-limited state in the *NRT2* TKO mutant is distinct from the nitrogen-deficient conditions caused by removal of nitrogen from the medium.

### Effects of elevated CO_2_ on metabolite levels in the shoot of the WT and the TKO_2-1/2/4 mutant

**Fig. 8:**
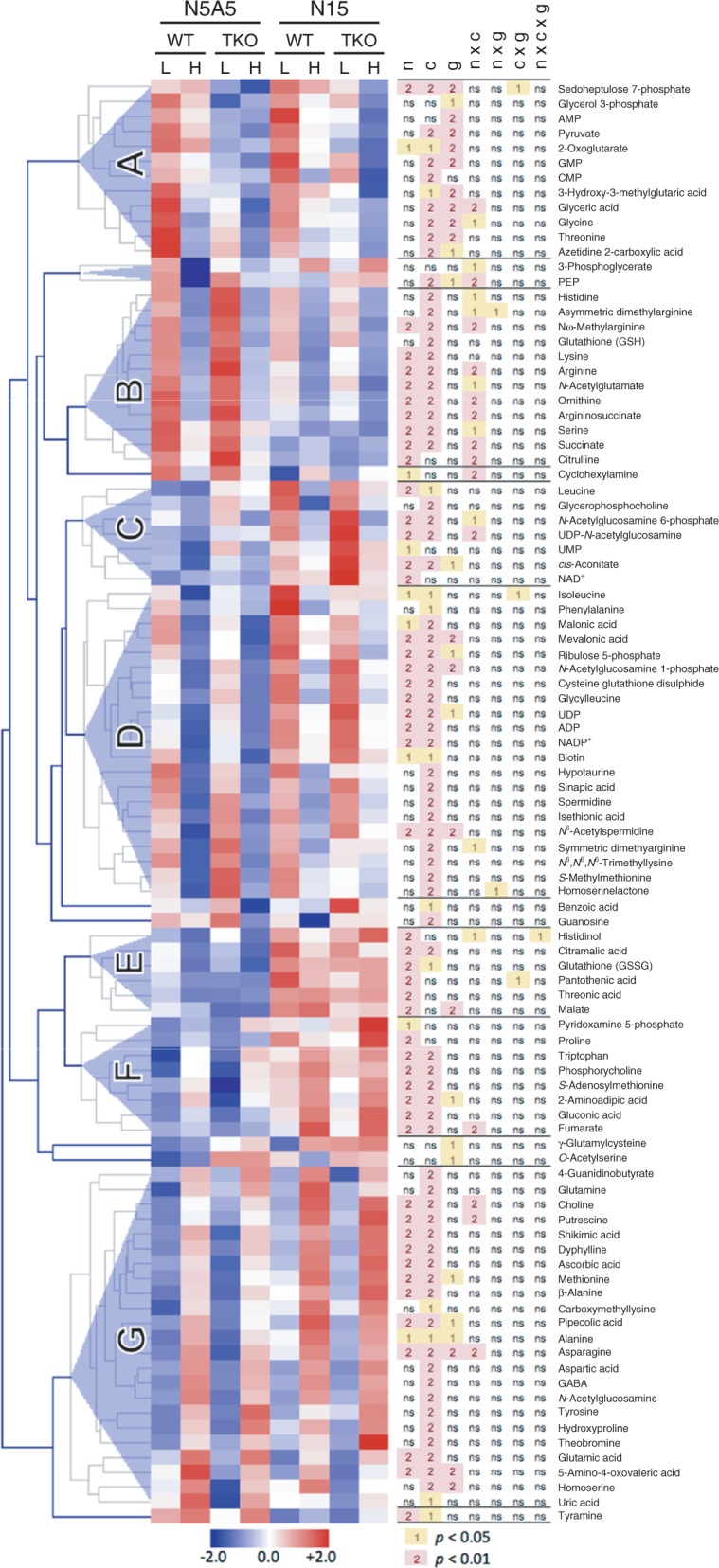
Heat map showing the metabolite profiles in the shoot of the WT and the TKO_2-1/2/4 mutant. Metabolite levels (average values from nine experimental replicates, *n* = 9) were normalized to Z-scores for each metabolite (blue–white–red heat map). Blue and red colors indicate a low and high metabolite level, respectively. Clustering was conducted based on the Euclidean distance for metabolites. Labels A–G indicate prominent clusters. Labels L and H indicate low (280 p.p.m.) and high (780 p.p.m.) CO_2_ conditions, respectively. The results of a three-way ANOVA for each metabolite are shown next to the heat map. Labels n, c and g indicate the analytical factors: CO_2_ level, nitrogen conditions and genotype, respectively. Labels ns, 1 and 2 denote not significant, *P* < 0.05 and *P* < 0.01 of the ANOVA, respectively.

**Fig. 9:**
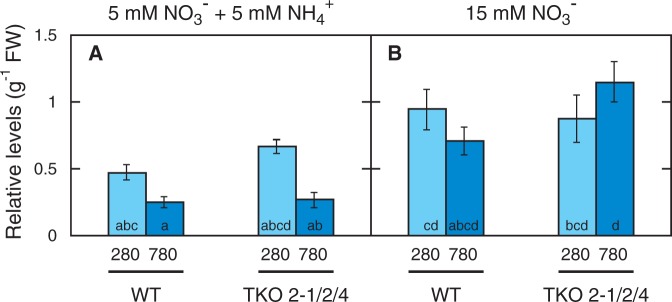
Effects of elevated CO_2_ on histidinol levels in shoots of the WT and the *NRT2* TKO mutant. Plants were grown on the N5A5 medium (A) or the N15 medium (B) under low (280 p.p.m.) or elevated (780 p.p.m.) CO_2_ levels for 10 d. Data shown are means ± SE from nine experimental replicates (*n* = 9). Different letters denote significant differences from a Scheffe’s multiple comparison test (*P* < 0.05) conducted for all data sets.

Changes of metabolite levels in the shoot of the WT and the TKO_2-1/2/4 mutant were analyzed by using capillary electrophoresis time-of-flight mass spectrometry (CE-TOFMS). Of the 130 metabolites identified on the basis of their accurate mass, 104 were detected in >90% of the samples examined (i.e. >63 out of the 72 samples from nine experimental replicates) and subjected to a three-way analysis of variance (ANOVA) to determine the factors affecting the metabolite levels. After excluding the seven metabolites that did not show a significant response to any of the three factors considered, the remaining 97 metabolites were subjected to a clustering analysis, which classified the metabolites into seven major clusters on the basis of their responses to the CO_2_ condition, nitrogen condition and the genotypes of the plant (A–G in Fig. 8). The ANOVA showed that the CO_2_ level (280 p.p.m. vs. 780 p.p.m.) was the most influential factor; it affected the levels of 82 metabolites including 17 proteinogenic amino acids (Fig. 8). Elevated CO_2_ had a negative effect for the majority of the metabolites, which formed the clusters A–D in Fig. 8. CO_2_ had a positive effect only with the metabolites in clusters F and G. ANOVA revealed that the nitrogen condition (nitrate and ammonium vs. nitrate only) affected 57 metabolites including 11 proteinogenic amino acids. In the presence of ammonium, levels of most of the affected metabolites were lower than those in the seedlings grown with nitrate as the sole nitrogen source. The metabolites that were increased in the presence of ammonium included the ornithine cycle metabolites (*N*-acetylglutamate, ornithine, citrulline, argininosuccinate and arginine) and positively charged amino acids (histidine and lysine), which were found to form cluster B. The positive effect of ammonium on the accumulation of the ornithine cycle metabolites was consistent with a previous report on mature Arabidopsis plants (Ferrario-Mèry et al. 2006). Most of the metabolites in this cluster were affected by the interaction of the nitrogen and CO_2_ conditions as revealed by the ANOVA (Fig. 8). Since the metabolite levels in this cluster were increased by ammonium and decreased by the elevated CO_2_, they were considered to reflect the N/C balance of the plant. It was therefore unexpected that the levels of these compounds in the TKO_2-1/2/4 mutant were not significantly different from the corresponding WT levels even when grown under 780 p.p.m. CO_2_ with nitrate as the nitrogen source, under which conditions the seedlings clearly developed the symptom of nitrogen limitation (Figs. 2–4). Although the three-way ANOVA showed that the genotype affected 28 metabolites including five amino acids, the changes in the metabolite levels ascribed to the genotype difference were observed mostly irrespective of the nitrogen and the CO_2_ conditions, as typically found in the metabolites in cluster A (Fig. 8). Histidinol, a precursor of histidine, was the only metabolite possibly affected by the interaction of the nitrogen conditions, CO_2_ and the genotype; levels of histidinol in the WT and in the mutant growing on the N5A5 medium were decreased by elevated CO_2_, whereas they were increased by elevated CO_2_ in the mutant growing on the N15 medium (Fig. 9). The similarity of the metabolome of the TKO mutant to that of the WT suggested that the seedlings can maintain the WT levels of most of the metabolites even under the constantly nitrogen-limited conditions.

## Discussion

In order constantly to limit the supply of nitrogen to the plant, the *A. thaliana* TKO mutant defective in the three *NRT2* genes, i.e. *NRT2.1*, *NRT2.2* and *NRT2.4*, was grown on a medium containing nitrate (15 mM) as the sole nitrogen source in this study. Despite the 50% lower nitrate content as compared with the WT (Fig. 3), the seedlings of the mutant showed no visible phenotype under 280 p.p.m. CO_2_ (Figs. 1, 2), indicating that the growth of the mutant as well as the WT was limited by CO_2_ availability and not by nitrogen availability. Under high CO_2_, the total shoot and root fresh weight in the mutant was 20% larger than that under the low-CO_2_ conditions (Fig. 2D, E), indicating that the increased CO_2_ availability stimulated the growth of the mutant, although the stimulation was not as obvious as in the WT, i.e. 68%. The low nitrate levels in shoots and roots of the mutant under these conditions (Fig. 3C, D) indicate that growth was retarded by slow assimilation of nitrate in the mutant, which was reflected in the high content of anthocyanin (Fig. 4B). Thus, nitrogen limitation was induced by growth of the mutant under elevated CO_2_.

In addition to the low nitrate content and high anthocyanin content, the mutant showed a decreased shoot/root ratio when grown under the high-CO_2_ conditions, which is thought to be another symptom of nitrogen limitation. It should be pointed out that the low shoot/root ratio resulted from the specific enhancement of root growth under high CO_2_. This is in contrast to the decrease in shoot/root ratio in the nitrogen-starved seedlings, which resulted mainly from the inhibition of shoot growth (Fig. 6). Therefore, the specific enhancement of root growth is considered a characteristic response to elevated CO_2_ in the seedlings under the constantly nitrogen-limited conditions. Another characteristic response of such plants is the increase in Chl content; while the nitrogen-starved plants have a markedly decreased Chl content (Fig. 6), the mutant in which nitrogen limitation was induced by elevated CO_2_ showed a significantly increased Chl content (Fig. 4). Reducing photosynthesis activity by decreasing Chl content is an important adaptive response to nitrogen deficiency (Peng et al. 2007). As opposed to the typical plant response to nitrogen deficiency, the photosynthetic capacity under elevated CO_2_ seems to be increased under the constantly nitrogen-limited conditions.

**Fig. 10:**
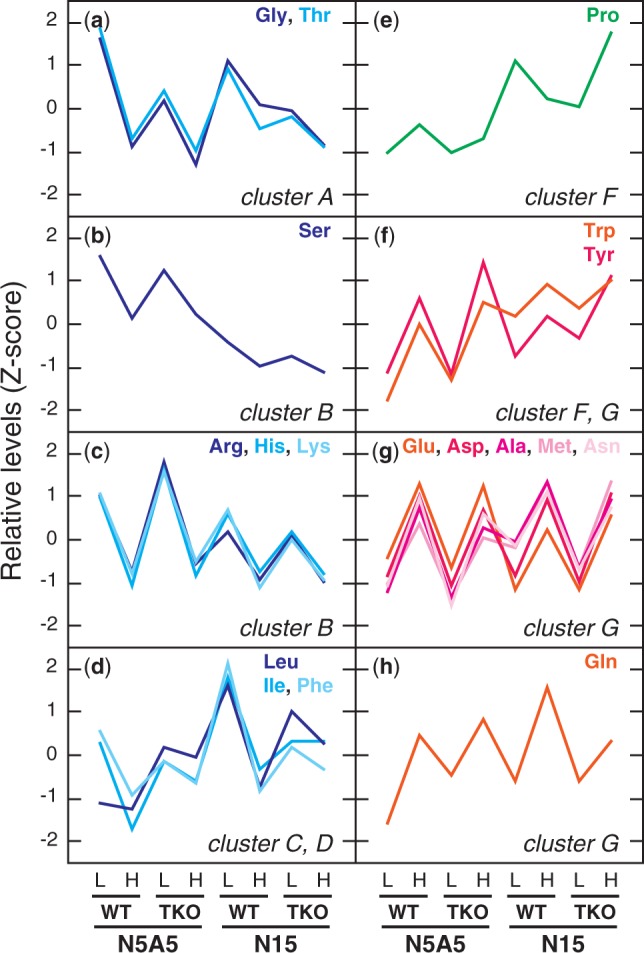
Effects of the CO_2_ and nitrogen conditions and the genotype on the levels of proteinogenic amino acids in the shoot. The Z-score profile of each amino acid is shown. L, 280 p.p.m. CO_2_; H, 780 p.p.m. CO_2_.

Li at al. (2008) showed that transfer of Arabidopsis plants in the rosette stage to high-CO_2_ conditions induced the response to nitrogen limitation. This was accompanied by an increase or decrease of the 17 amino acids examined, 11 of which were shown to change in the same way after withholding of nitrogen from a hydroponic culture of Arabidopsis (Krapp et al. 2011), confirming that the transition to a high-CO_2_ environment did bring about the conditions of nitrogen limitation. In our study, comparison of the seedlings growing under high CO_2_ and low CO_2_ showed that in both the WT and the *NRT2* TKO mutant, glycine, threonine, serine, arginine, histidine, lysine, leucine, isoleucine and phenylalanine were decreased by high CO_2_ (Fig. 10, panels a–d), while tryptophan, tyrosine, alanine, methionine, aspartate, glutamate, asparagine and glutamine were increased (Fig. 10, panels f–h). These results are distinct from those reported by Li et al. (2008), where the levels of nine amino acids (alanine, aspartate, asparagine, glutamate, glutamine, lysine, histidine, phenylalanine and tyrosine) were affected by elevated CO_2_ in the opposite direction. The effects of high CO_2_ observed in this study are rather similar to those found in tobacco plants by comparing the high-CO_2_- and low-CO_2_-grown plants (Geiger et al. 1998), with seven amino acids including aspartate, asparagine, glutamate, glutamine and histidine being changed in the same way, while most other amino acids were not affected by CO_2_ in tobacco. These results confirm that the state of plants growing under the high-CO_2_-induced, constantly nitrogen-limited conditions is distinct from that during transition to the nitrogen-limited conditions.

Although the metabolite profile showed that the level of histidinol, a precursor of histidine, was characteristically altered in the mutant growing under the 15 mM 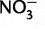 conditions in elevated CO_2_ as compared with that in the WT (Fig. 9), there was no difference in the histidine level between the mutant and the WT (Fig. 10). Since the HISN7 enzyme catalyzing the dephosphorylation of histidinol-P to histidinol shows a broad substrate range in vitro, including d-inositol-1(or 3)-P and l-galactose-1-P (Torabinejad et al. 2009), the presence of additional potential links between histidine biosynthesis and other metabolic pathways has been suggested (Petersen et al. 2010). These metabolic pathways, which remain to be demonstrated in vivo, might respond specifically to elevated CO_2_ in the mutant in the constantly nitrogen-limited state.

In summary, a constantly nitrogen-limited state was established when the *NRT2* TKO mutant was grown under high CO_2_ on nitrate-containing medium. The response of the mutant seedlings to this state included specific enhancement of root growth and the increase of Chl content, which would help maximize the capacity for nitrogen assimilation and photosynthesis, respectively, under the CO_2_-enriched and nitrogen-limited conditions. Although the accumulation of anthocyanin indicates that the plant is experiencing the stress of nitrogen limitation, the apparently normal metabolite profile as compared with the WT suggests that the plant can maintain normal metabolism under such conditions. Since the influence of elevated CO_2_ on the transcriptome, metabolome and nitrogen assimilation is affected by the developmental stage of the plant (Geiger et al. 1998, Kaplan et al. 2012), further studies are required to determine the responses to elevated CO_2_ of the mature plants in the constantly nitrogen-limited state.

## Materials and Methods

### Plant materials and growth conditions

The media used for plant growth were modifications of half-strength Murashige and Skoog medium, containing 1.5 mM CaCl_2_, 0.75 mM MgSO_4_, 0.625 mM KH_2_PO_4_, 55 µM Na_2_EDTA, 50 µM FeSO_4_, 2.5 µM KI, 50 µM H_3_BO_3_, 50 µM MnSO_4_, 15 µM ZnSO_4_, 0.5 µM Na_2_MoO_4_, 0.05 µM CuSO_4_ and 0.05 µM CoCl_2_. To the basal medium, 15 mM KNO_3_ was added to prepare a medium containing nitrate as the sole nitrogen source (N15 medium). KNO_3_ (5 mM), 2.5 mM ammonium succinate and 10 mM KCl were supplemented to the basal medium for preparation of a medium containing both nitrate and ammonium (N5A5 medium). Seeds of *A. thaliana* WT (Col-0) and the triple mutant defective in *NRT2.1*, *NRT2.2* and *NRT2.4* (Kiba et al. 2012; designated TKO_2-1/2/4) were surface-sterilized and sown on medium supplemented with 0.8% agar, 1% sucrose and 5 mM MES-KOH (pH 5.8). Plants were grown in controlled environmental growth chambers (NIPPON MEDICAL & CHEMICAL INSTRUMENT CO., LTD) with a photosynthetic flux of 200 µmol m^−2 ^s^−1^ under a 16 h light/8 h dark cycle at 22°C with 70% relative humidity. The CO_2_ concentration was kept at 280 p.p.m. (low CO_2_) or 780 p.p.m. (elevated CO_2_).

For preparation of nitrogen-starved plants, seedlings were grown for 7 d on N5A5 medium supplemented with 0.2% gellan gum, 1% sucrose and 5 mM MES-KOH (pH 5.8) in a Sanyo growth chamber under ambient CO_2_ concentrations and then transferred to a nitrogen-free medium containing 15 mM KCl (–N conditions) or the N5A5 medium (+N conditions, control) and grown for 3 d.

### Determination of nitrate, total nitrogen and total carbon in plants

For determination of nitrate, seedlings were separated into shoots and roots, weighed, and collected in microcentifuge tubes. Distilled water amounting to 49 times the weight of the tissue was added to each tube and autoclaved at 110°C for 20 min. After centrifugation, the supernatant was used for measurement of the nitrate concentration by a flow injection analyzer (PFA-310NO, FIA Co.). For determination of total contents of nitrogen and carbon, shoots from 10–30 plants from each treatment were pooled, dried and ground to powder. The samples were weighed and the carbon and nitrogen content was measured by a CN corder (Macro Corder JM1000CN, J- Science Lab.), using hippuric acid as a standard.

### Determination of pigment contents in shoots

Total anthocyanins in shoots were extracted and determined as described by Laby et al. (2000) with minor modifications. Weighed shoots were collected in microcentrifuge tubes, to which 0.5 ml of methanol–1% HCl was added. After incubation at 4°C for 16 h, the debris was removed by centrifugation, and absorbance at 530 and 657 nm of the supernatant was measured and used to calculate total anthocyanin content. Chl in shoots were extracted and determined as described by Porra et al. (1989) with minor modifications. Weighed shoots were homogenized in 0.5 ml of 80% acetone and, after centrifugation, absorbance of the supernatant at 646.6 and 663.6 nm was measured and used to calculate Chl content.

### Quantitative reverse transcription–PCR

Total RNA was extracted using an RNeasy Plant Mini Kit (QIAGEN) according to the manufacturer’s instructions. Total RNA concentrations were determined by UV spectrophotometry. First-strand cDNAs were reverse transcribed by an oligo(dT) primer using the PrimeScript™ II 1st strand cDNA Synthesis Kit (TAKARA). An aliquot of the first-strand cDNA mixture, obtained from 100 ng of total RNA, was used as a template for the PCR. PCR was performed on a LightCycler instrument (Roche) with the LightCycler-FastStart DNA Master SYBR Green kit (Roche) according to the manufacturer’s instruction. The gene-specific primers were designed to produce 185, 109 and 72 bp DNA fragments from the cDNAs of *CHLI1* (At4g18480), *HEME2* (At2g40490) and *EF1A4α* (At5g60390) cDNA used as an internal control, respectively. The sequences of the primers are as follows. *CHLI1*: forward primer, CCGGCGAGGTTTATCT; reverse primer, TTTGTAAGTGTCACGGAAAT. *HEME2*: forward primer, TCAATCAGCTGCCGACGTT; reverse primer, CGGCTTCATTGTTCACCTCA. *EF1A4α*: forward primer, CTGGAGGTTTTGAGGCTGGTAT; reverse primer, CCAAGGGTGAAAGCAAGAAGA.

### Metabolome analysis

Approximately 50 mg of 10-day-old shoots from each of the eight conditions tested was weighed and homogenized in 0.5 ml of methanol containing 100 µM PIPES and l-methionine sulfone as internal standards. Then 0.5 ml of chloroform and 0.2 ml of distilled water were added to the homogenate and mixed. After centrifugation, 0.4 ml of the water–methanol layer was filtered through a Millipore 5 kDa cut-off filter and dehydrated. The extracted metabolites were dissolved in 0.1 ml of distilled water and analyzed by CE-TOFMS (Agilent Technologies). For determination of anionic compounds, separations were performed at 30 kV on a fused silica capillary (100 cm × 50 µm) with 20 mM ammonium formate (pH 10) as a running buffer and 50% (v/v) methanol as a sheath liquid. For determination of cationic compounds, separations were performed at 27 kV on a fused silica capillary (100 cm × 50 µm) with 1 M formic acid (pH 1.9) as a running buffer and 50% (v/v) methanol as a sheath liquid. Metabolites in the extract were identified by their *m/z* ratio and calculated as a relative value compared with the internal standard. The average level of each metabolite from nine biological replicates was calculated for each of eight conditions and converted to Z-scores normalizing the rate of change among the eight conditions. The Z-scores of metabolites were aligned by a hierarchical clustering analysis with an Euclidian distance metric, and visualized as a heat map representation using MeV ver. 4.8, TM4 software (Dana-Farber Cancer Institute) (Saeed et al. 2003). The clusters were configured in 2.3 of the distance threshold.

### Statistical analysis

All the statistical analyses were conducted with SPSS Statistics software (IBM).

## Funding

This work was supported by the Ministry of Education, Culture, Sports, Science and Technology, Japan [a Grant-in-Aid for Scientific Research in Innovative Areas (No. 21114003)].

## Disclosures

The authors have no conflicts of interest to declare.

## Abbreviations

ANOVA: analysis of variance
CE-TOFMS: capillary electrophoresis time-of-flight mass spectrometry
NRT: nitrate transporter
TKO: triple knockout
WT: wild type
